# Thermal inactivation of *Salmonella* on chicken wings cooked in domestic convection and air fryer ovens

**DOI:** 10.1111/1750-3841.16230

**Published:** 2022-06-28

**Authors:** Carmen Cano, Xinyao Wei, Cyril A. Etaka, Byron D. Chaves

**Affiliations:** ^1^ Department of Food Science and Technology University of Nebraska‐Lincoln Lincoln Nebraska USA

**Keywords:** Salmonella, chicken parts, thermal inactivation, air fyer, convection oven

## Abstract

Chicken wings are among the most popular poultry products for home and foodservice consumption. Poultry products must be handled and cooked safely to decrease the risk of foodborne salmonellosis for consumers. This study aims to validate the use of domestic appliances (convection and air fryer ovens) for the thermal inactivation of *Salmonella* on chicken wings. Wings (*n* = 3, 46.5 ± 4.3 g) were inoculated with a five‐strain cocktail of *Salmonella* (ca. 8 log_10_ CFU/wing) and cooked in a convection oven (179.4°C) or an air fryer (176, 190, or 204°C) for 2, 5, 10, 15, 20, 22, or 25 min. Thermocouples recorded temperature profiles of wings and appliances. *Salmonella* counts were determined on XLD agar for rinsates (100 ml/sample), and rinsates were enriched to recover bacteria below the limit of quantification. The recommended internal cooking temperature (73.8°C) was achieved after a range of 7.5 to 8.5 min in both appliances. *Salmonella* counts were reduced by 6.5 log_10_ CFU/wing when this temperature was achieved. Cumulative lethality (F‐value) calculations predicted a 9‐log reduction after 7.0 to 8.1 min of cooking. However, sample enrichments tested positive for *Salmonella* for all cooking times below 22 min. Ultimately, cooking at the temperature–time combinations recommended by manufacturers and online recipes helped achieve complete microbial elimination in both appliances. This study contributes to the validation of home cooking methods to ensure consumer safety.

## 1 INTRODUCTION

Many consumers believe that meals prepared outside the home are more likely to cause foodborne illness; however, between 1997 and 2017, there were more than 2500 foodborne disease outbreaks and 110 deaths from food sources in private homes, as reported in the National Outbreak Reporting System for foodborne outbreaks in a private home setting in this time period (Centers for Disease Control & Prevention, 2018). From these cases, 80 outbreaks and one death were attributed to the consumption of poultry products contaminated with *Salmonella* (setting: private home/residence, etiology: *Salmonella*, food/ingredient: chicken) (Centers for Disease Control & Prevention, 2018). In comparison, there were 132 outbreaks and two deaths in this period that are attributed to poultry products contaminated with *Salmonella* consumed in a restaurant setting (setting: fast‐food, buffet, sit‐down or other, etiology: *Salmonella*, food/ingredient: chicken) (Centers for Disease Control & Prevention, 2018). Preventing temperature abuse, minimizing cross contamination, and avoiding consumption of undercooked poultry products are the most effective measures to reduce the probability of poultry‐borne foodborne illness (Collineau et al., 2020).

The United States Department of Agriculture Food Safety and Inspection Service recommends cooking poultry to a minimum internal temperature of 74°C (165°F) and using a properly calibrated food thermometer to verify that internal temperature (U.S. Department of Agriculture, Food Safety & Inspection Service, 2019). However, observational studies of chicken preparation at home have found that consumers tend to overestimate how often they comply with safe food handling practices (Barrett et al., 2020; Bruhn, 2014). An at‐home chicken preparation study, carried out with 120 volunteers who prepared chicken dishes in their homes, found that nearly half of volunteer participants owned a thermometer, but only about one‐third knew the USDA‐recommended cooking temperature for poultry. Additionally, while the majority reported using thermometers when cooking whole chicken, only one‐third would use them when cooking chicken parts (Bruhn, 2014). When cooking a chicken dish as part of the study, only 5% of volunteers (6/120) used a food thermometer to check for doneness without prompting from the researcher (Bruhn, 2014). Roughly half of the chicken dishes were considered done when the end point temperature was below 74°C (grilled chicken: 17/33 dishes, fried chicken: 19/46, roasted: 9/33, boiled: 2/7, and pressure cooked: 0/1). Additionally, a study of 474 poultry recipes found that the most common methods to determine doneness were cooking time (94%) and visual inspection (40%), while only 33.7% provided a specific temperature for doneness (Chambers et al., 2018). Consumers following these recipes may not feel the need to verify the internal temperature of the product if it is not in the recipe. These findings reveal a gap in food safety practices in domestic settings that may compromise the microbiological safety of the product. Therefore, it is important to validate the cooking times and temperatures in these recipes (Chambers et al., 2018).

Several cooking methods are used to prepare chicken wings at home, including baking, frying, and grilling. Air frying is a cooking method in which hot air circulates uniformly around the food, achieving similar physical and chemical changes as hot oil frying with less or no gain of oil (Andrés et al., 2013). This method has become popular recently as air fryers are commercially available and consumers look for healthier alternatives to fried foods (Zaghi et al., 2019). Air fryers achieve high transfer rates between air and the food, using an air blower, high convective rates, radiative heat transfer, and especially designed cooking chambers (Teruel et al., 2015). However, air frying requires a longer cooking time compared to deep frying, as air has a lower heat transfer coefficient than oil (Zaghi et al., 2019). This also results in a slower formation of the crust associated with fried products and greater loss of water mass in air‐fried products compared to hot oil‐fried products (Andrés et al., 2013). Reported recommended temperatures for air frying chicken wings range between 176°C and 204°C (350 to 400°F). Given the novelty of air fryers as domestic kitchen appliances, few studies have explored pathogen inactivation using this technology in a domestic setting (Rao et al., 2020). Therefore, the goal of this study was to determine the thermal lethality of *Salmonella* on chicken wings cooked in a conventional convection oven or an air fryer. The results of this study provide validated cooking times and temperatures that may be added to cooking recipes to ensure thermal destruction of *Salmonella* in poultry products when using a dry‐heat method of cooking, such as chicken wings that are baked in a convection oven or an air fryer.

## 2 MATERIALS AND METHODS

### 2.1 Sample preparation and inoculation

Trays of fresh chicken wings, which included both drums and flats (also known as drummets and wingettes), were purchased from a grocery store in Lincoln, Nebraska. The average weight of chicken wing was 46.5 ± 4.3 g. Samples were kept at 4°C and used within 72 h of purchase. Preliminary assessments showed low numbers of aerobic plate counts (< 2 log_10_ CFU/wing) and *Salmonella* (< 2 log_10_ CFU/wing) on the brand of chicken wings purchased. No differences were found between drums and flats. Five poultry products‐borne *Salmonella* strains from the University of Nebraska‐Lincoln Food Processing Center collection, namely Branderup (*n *= 1), Enteritidis (*n* = 2), Hadar (*n* = 1), and Typhimurium (*n* = 1) were used for sample inoculation. Strains were stored in tryptic soy broth (TSB, Remel, Lenexa, KS) with 20% glycerol at −80°C. For the experiment, isolates were removed from frozen storage, and each strain was inoculated into 10 ml TSB and incubated staticly at 37°C for 18–24 h. For each strain, 1 ml of culture was transferred to 200 ml of TSB and incubated staticly at 37°C for 18 to 24 h. After this incubation, the concentration of each strain in the TSB was as follows: Branderup, 8.4 ± 0.1 log_10_ CFU/ml; Enteritidis strain #1, 8.7 ± 0.1 log_10_ CFU/ml; Enteritidis strain #2, 8.6 ± 0.1 log_10_ CFU/ml; Hadar, 8.6 ± 0.1 log_10_ CFU/ml; and Typhimurium, 8.6 ± 0.1 log_10_ CFU/ml. The five cultures were mixed in a sterile stainless‐steel tub inside a biosafety cabinet. The resulting cocktail had a concentration of 8.6 ± 0.2 log_10_ CFU/ml. Chicken wings were immersed in the inoculated broth for 30 s without mixing. Forty‐five chicken wings (mean 46.5 ± 4.3 g) were inoculated per replicate and each appliance/temperature combination. The combined chicken weight added to each inoculated broth was 2094.2 ± 4.3 g. Wings were then placed on sterile metal racks in a biosafety cabinet for 20 min to allow for bacterial attachment. Three chicken wings from each replicate were reserved to estimate the starting inoculation level. Preliminary studies confirmed that a consistent level of inoculation (8.5 ± 0.1 log_10_ CFU/wing) was achieved with this method.

### 2.2 Household appliances

For this study, two household appliances that use dry heat for cooking were selected. The air fryer (Emeril Lagasse Pressure Air Fryer Plus, Model Y6D‐AF‐36B, Emeril Everyday, Tristar Products Inc., Santa Rosa Beach, FL) had a capacity of 6 quarts (5.68 L) and a temperature range of 82°C to 204°C (180°F to 400°F). The tabletop convection oven (Flavor Wave Oven Deluxe, Thane Housewares, La Quinta, CA) had a capacity of 15.7 L, and a temperature range of 88°C to 204°C (190°F to 400°F). The tabletop convection oven was chosen for comparison as its design is very similar to that of an air fryer and its use has been discussed in previous literature (Murphy, Johnson, Duncan, et al., 2001; Murphy, Johnson, Marks, et al., 2001).

### 2.3 Thermal treatment of chicken wings

For each experimental replicate, chicken wings (nine pieces, average combined weight 418 ± 38 g, weight range 346.6 g to 524 g) were placed either in a tabletop convection oven or an air fryer, described above. This number of wings was chosen to cover most of the surface of the air fryer product rack without overcrowding. Type T thermocouples (Omega, Norwalk, CT, USA) were placed in the center of three different chicken wings to measure the internal product temperature. The chicken wings were located as follows: one in the center of the rack and two in the outer diameter of the rack. The initial internal product temperature was recorded once the wings were placed in the appliance. A fourth thermocouple was placed close to the center of the appliance, without touching the wings, to measure the air temperature inside the appliance. The thermocouples were plugged into a data logging system (TC‐08 8 channel USB data acquisition module and corresponding logging software, Omega) set to record the temperature every 15 s. Cooking times can be set manually on both appliances. The convection oven was set to 179°C/355°F. The air fryer was set at the manufacturer's suggested temperature for air frying any type of food (176°C/350°F). Neither manufacturer recommended preheating the appliance before cooking the wings. Afterward, the air fryer treatment was repeated with two other temperatures (190°C/375°F and 204°C/400°F), so that three temperatures were tested for the air fryer. To protect the consumer from the hot appliance, this air fryer has 2 min of appliance cool down before the appliance can be opened and the food can be removed. Chicken wings were cooked for 2, 5, 10, 15, 20, 22, or 25 min. A fresh set of nine raw chicken wings (three non‐inoculated wings with thermocouples and six inoculated wings) was used for each cooking time. Three wings from each cooking time were removed with sterile tongs and placed individually in sterile 55 oz nonfiltered sample bags on ice for at least 5 min.

### 2.4 Microbiological analysis

One hundred milliliters of buffered peptone water (BPW, Sigma‐Aldrich, St. Louis, MO, USA) were added to each bag, and the samples were shaken manually for 60 s to detach bacteria. This method was adapted from previous literature (Scott et al., 2015). Appropriate decimal serial dilutions were prepared in 0.1% BPW and plated onto Xylose Lysine Deoxycholate agar (XLD, BD, Franklin Lakes, NJ), then incubated at 37°C for 18 to 24 h. In preliminary studies, the dilutions were also plated onto tryptic soy agar (TSA), and later overlaid with XLD, to recover possible injured cells. The difference in counts between XLD plates and TSA+XLD plates was 0.1 log_10_ CFU/ml rinsate or less, so the overlay method was not considered necessary and a enrichment step was added instead for information on injured cells and uninjured cells below the limit of quantification. The experiment was repeated three times with fresh inocula and chicken samples. The results were recorded as log_10_ CFU/chicken wing. The limit of quantification was 1 CFU/ml rinsate, which corresponds to 100 CFU/chicken wing or 2 log_10_ CFU/chicken wing. Additionally, sample rinsates were incubated for 18 to 24 h at 37°C. For samples with counts below the limit of quantification, enrichments were streaked onto XLD agar and incubated at 37°C for 18 to 24 h. Plates with typical *Salmonella* colonies (opaque, yellow to red colonies with black centers) were recorded as *Salmonella* positive.

### 2.5 Data analysis

Survivor curves were prepared for each cooking method by plotting log_10_ CFU/chicken wing versus heating time. The expected cumulative lethality (F‐value) for each appliance and temperature combination was calculated based on D‐values and Z‐values for *Salmonella* in ground chicken taken from Murphy et al. (2004), using the equation:

(1) $$F_{o}=\int_{0}^{t}10^{\frac{T\left(t\right)-T_{R}}{z}}dt$$

where *F*_0_ is the cumulative lethality in log_10_ CFU at time t (min), *T*(*t*) is the temperature at time t, *T_R_* is the reference temperature (62.5°C), and *z* is the z value (5.34°C) for *Salmonella* in ground chicken meat. Log_10_ D‐values were plotted versus their corresponding temperatures on Microsoft Excel^®^, and a linear regression was found. The z‐value (5.336) was obtained by finding the inverse of the linear regression's slope. The reference temperature was set at 62.5°C because this temperature was used when establishing the D‐values. The F‐value was calculated for every 15‐s time point on chicken wing internal temperature profiles. A cumulative F‐value at each time point was calculated by adding up the lethality values for all previous time intervals. The time point that corresponded to a 7‐log reduction and a 9‐log reduction for each appliance, and temperature combination was determined. These reductions were chosen based on USDA‐FSIS requirements for ready‐to‐eat poultry products and the initial inoculation level of the chicken wings (U.S. Department of Agriculture, Food Safety & Inspection Service, 2017). The time points for 7‐log and 9‐log reductions were compared using single‐factor ANOVA on Microsoft Excel^®^, and differences were deemed significant at a 5% probability level.

## 3 RESULTS AND DISCUSSION

**FIGURE 1:**
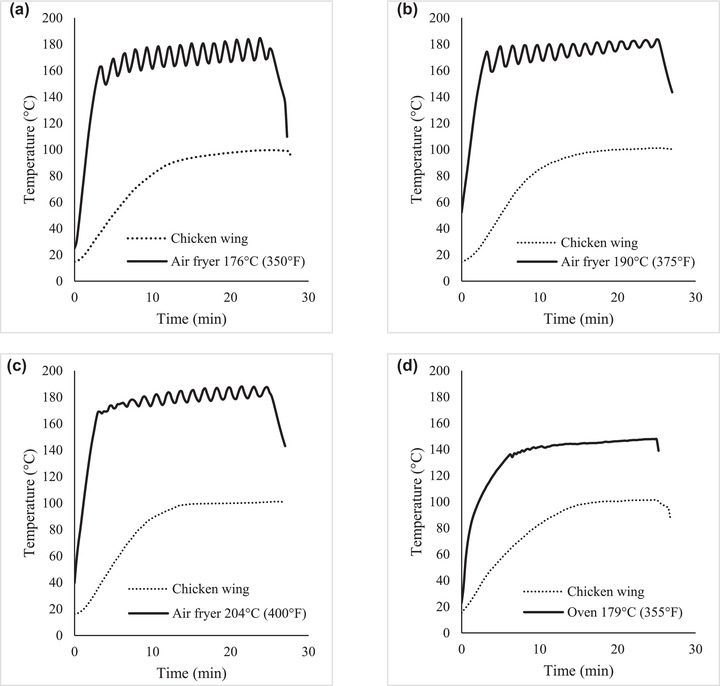
Thermal profile of chicken wings during cooking in (a) air fryer set at 176°C (350°F), (b) air fryer set at 190°C (375°F), (c) air fryer set at 204°C (400°F), and (d) convection oven set at 179°C (355°F). The profiles represent three trials, and three chicken wings were monitored per trial. Chicken wings were located: one in the center of the appliance and two on opposite sides, and their temperatures were averaged. The appliance thermal profile was measured with one thermocouple located in the middle of the appliance

The average initial temperature of the chicken wings was 13.7 ± 4.5°C (56.6 ± 8.1°F). The USDA‐FSIS‐recommended internal cooking temperature for poultry is 73.8°C (165°F), which was achieved after 7.7 ± 1.2 min in the convection oven set at 179.4°C (355°F), and after 8.2 ± 1.5, 8.6 ± 1.8, and 8.7 ± 2.1 min in the air fryer set at 176, 190, and 204°C (350, 375, and 400°F), respectively. Nominally, the temperature in both appliances was set to similar values: 179.4°C (355°F) for the convection oven and 176.6°C (350°F) for the air fryer. However, the average measured temperature inside the appliances differed significantly (*p* < 0.001) by 29.0°C. The average temperature in the convection oven was 142.5°C (288.5°F) with a maximum of 147.9°C (298.2°F), while the average temperature in the air fryer was 171.5°C (340.7°F), with a maximum of 184.7°C (364.5°F). This may be due to the construction of the appliances, as it was noticed that the air fryer was more insulated than the convection oven. Average and maximum temperatures inside the air fryer rose as the set temperature was increased (Figure 1). The air fryer set at 190.5°C (375°F) and 204.4°C (400°F) had average temperatures of 175.9°C (348.6°F) and 180.0°C (356°F), with maximum values of 183.7°C (362.7°F) and 187.9°C (370.2°F), respectively. It is worth noting that neither the average nor the maximum appliance temperatures reached the nominal set temperatures. Despite the differences in appliance temperature, the chicken wings achieved a maximum internal temperature of 99°C (210.2°F) in both appliances after 16.8 ± 2.3 min in the convection oven set at 179.4°C (355°F), and 20.0 ± 2.4 min in the air fryer set at 176.6°C (350°F), 17.8 ± 2.1 min set at 190.5°C (375°F), and 16.7 ± 2.3 min set at 204.4°C (400°F). Air fryers are designed to provide extremely high heat transfer rates, so high product temperatures were expected (Teruel et al., 2015). It is possible that the larger size of the convection oven allowed for faster heat transfer to the chicken wings.

In a study by Rao et al. (2020), a similar thermal inactivation experiment was performed using chicken strips in an air fryer, a toaster oven, a conventional oven, and a deep fryer. Their set temperature for the air fryer was 204°C (400°F) and, similarly to this study, the appliance failed to achieve the set temperature on the dial (maximum temperature 201°C/393.8°F) (Rao et al., 2020). Despite the higher cooking temperature, the time required for chicken strips to reach an internal temperature of 74°C (165°F) was similar (8.5 ± 1.5 min) to that in the current study (Rao et al., 2020). The chicken strips (40 g) were similar in size to the chicken wings in this experiment (46.5 ± 4.3 g); however, they were cooked from frozen (approximately −20°C), which may explain the similarity in cooking time despite the higher appliance temperature (Rao et al., 2020). Murphy, Johnson, Marks, et al. (2001) processed ground chicken breast patties (approximately 30 g each) in a convection oven at different air temperatures, ranging from 163°C to 218°C (325°F to 424°F). At 177°C (350.6°F), the heating time required to achieve an internal temperature of 75°C (167°F) was close to 20 min, which is much longer than the time observed in this study. At 190°C (374°F), the heating time needed was also longer than in this study, as the chicken patties required around 15 min to reach 75°C (167°F). Heating time could be influenced by the difference in the sample composition (ground chicken compared to bone‐in whole muscle) or the differences in appliance construction. Relative humidity inside air convection ovens can greatly influence product internal temperature, and higher relative humidity results in shorter cooking times due to its high conductivity (Murphy, Johnson, Duncan, et al., 2001). The initial temperature of the patties (4°C) was also lower than in this study (13.7 ± 4.5°C/56.6 ± 8.1°F).

**FIGURE 2:**
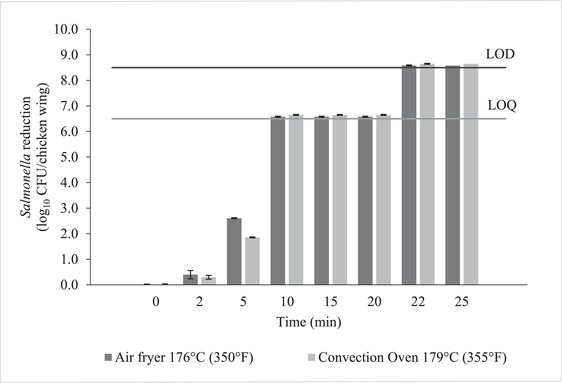
*Salmonella* reduction (log_10_ CFU/chicken wing) in chicken wings during thermal treatment in a convection oven set at 179°C (355°F) or an air fryer set at 176°C (350°F). Actual appliance temperatures were lower than the set points. Error bars represent standard error of the mean. LOD line represents the limit of detection and LOQ line represents the limit of quantification

**FIGURE 3:**
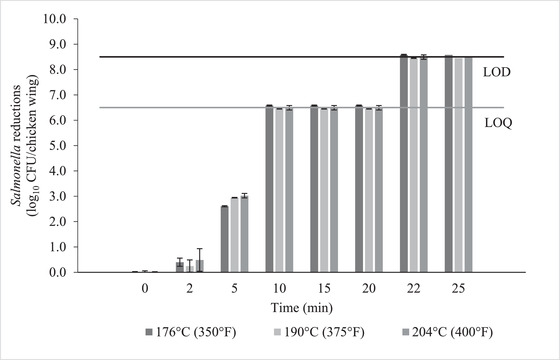
*Salmonella* reduction (log_10_ CFU/chicken wing) in chicken wings during thermal treatment in air fryer set at three different temperatures (176°C, 190°C, and 204°C). Actual appliance temperatures were lower than the set points. Error bars represent standard error of the mean. LOD line represents the limit of detection and LOQ line represents the limit of quantification

Reductions of *Salmonella* counts on chicken wings are shown in Figures 2 and 3. Initial counts were 8.5 ± 0.1 log_10_CFU/chicken wing. Both appliances were capable of reducing the bacterial counts to below the limit of quantification. *Salmonella* counts decreased drastically between 5 min and 10 min of cooking in both appliances, which coincides with the reaching of 74°C (165°F) internal temperature, as discussed above. When comparing appliances, there was a higher initial reduction in counts for the air fryer at 2 and 5 min compared to the convection oven (Figure 2). This may be due to the additional 2 min of holding time added by the air fryer as its heating element cools down, as well as the higher internal temperature inside the air fryer. Figure 3 compares the three different treatment temperatures in the air fryer. There was no a significant difference in reductions between the treatments, which could be due to the small difference in actual appliance temperature as described above.

Counts for samples cooked for 10 min and longer were below the limit of quantification (2 log_10_ CFU/chicken wing) for all treatments. The use of selective media can make it harder to recover sublethally injured cells, so an enrichment step was added for these samples (Juneja, 2007). Enriched samples continued to test positive for *Salmonella* for cooking times below 22 min, as cells might be present below the limit of quantification. Additionally, cells may have been injured but not inactivated during shorter cooking times. This has been reported before by Juneja (2007) by plating heat‐treated chicken samples on both XLD and TSA plates. For the air fryer at 176.6°C (350°F), this coincides with the time needed for the chicken wings to achieve an internal temperature of 99°C (210.2°F, t = 20.0 ± 2.4 min). Chicken wings in the convection oven achieved that internal temperature much sooner, but this did not decrease the required cooking time. It is possible that the internal temperature as measured did not reflect the temperature of the entire chicken wing, since the temperature may not be uniformly distributed due to shape and composition differences (Juneja et al., 2001; Rao et al., 2020). Rao et al. (2020) were able to detect *Salmonella* on inoculated chicken strips cooked for 10 min at 204°C (399.2°F). The chicken strips reached an internal temperature of 98°C (208.4°F), and *Salmonella* was detected on all nine of the strips, with average reductions of 4.3 log MPN/g. Murphy, Johnson, Marks, et al. (2001) also detected *Salmonella* on chicken patties cooked to a center temperature of 80°C (176°F). The survival of *Salmonella* could be explained by temperature gradients throughout the product, as well as the high humidity of the environment during cooking (Murphy, Johnson, Marks, et al., 2001).

The time to achieve cumulative lethality (F‐value) for the different treatments is presented in Table 1. The time for a 7‐log reduction was estimated, since this is the regulatory requirement for ready‐to‐eat poultry products (U.S. Department of Agriculture, Food Safety & Inspection Service, 2017). The time for a 9‐log reduction was also calculated to account for the initial inoculation level of the chicken wings. As shown in Table 1, the calculated time for a 7‐log reduction ranged from 6.9 ± 1.0 min to 7.9 ± 2.0 min for the different treatments, and there was no significant differences between treatments (*p* = 0.296). The lack of difference could be explained by the high variation in temperatures observed between replications, which is reflected in the high standard deviation of the F‐values. Additionally, D‐values at high temperatures are in the order of seconds, which results in minimal time differences between each treatment temperature (Murphy et al., 2004). These predictions match closely with the times to reach a 74°C internal temperature and with the *Salmonella* counts being lower than the level of quantification. The calculated times for a 9‐log reduction were almost the same as for a 7‐log reduction, which does not match the observed recovery of *Salmonella* from wings. The times for a 9‐log reduction were also not significantly different between treatments (*p* = 0.301). Additionally, there was no significant difference between the time needed for a 7‐log reduction and the time needed for a 9‐log reduction for any of the treatments (air fryer set at 176°C: *p* = 0.905; air fryer set at 190°C: *p* = 0.910; air fryer set at 204°C: *p* = 0.861; oven set at 179°C: *p* = 0.808). The lack of difference in cumulative lethality time can be explained by the high temperatures achieved in this study. As treatment temperatures increase, D‐values decrease and the time required to go from a 7‐log to a 9‐log reduction would become minimal (Murphy et al., 2004).

**TABLE 1:** Time (min ± standard deviation) required for reductions of *Salmonella* counts in chicken wings based on the expected cumulative lethality

Appliance	Temperature (°F/°C)	Time for a 7‐log reduction (min)	Time for a 9‐log reduction (min)
Air fryer	350/176	7.6 ± 1.5^a^	7.7 ± 1.4^a^
	375/190	7.8 ± 1.5^a^	7.8 ± 1.6^a^
	400/204	7.9 ± 2.0^a^	8.1 ± 2.0^a^
Convection oven	355/179	6.9 ± 1.0^a^	7.0 ± 1.1^a^

*Note*: Within a column, values with the same letter superscript are not significantly different at a 5% probability level.

Survival of *Salmonella* may be explained by sample or treatment factors. The calculations were based on D‐values for ground chicken thigh meat in homogeneous 10‐g packages, where the loss of moisture was minimal (Murphy et al., 2004). Chicken wings are a more complex sample, with meat and skin surfaces, as well as higher potential for moisture loss. A comparison of thermal inactivation of *Salmonella* on whole and ground turkey breasts found that the inactivation rate was greater in ground samples than in whole muscle samples (Tuntivanich et al., 2008). Similar results have been observed in beef samples, which suggests that the physical state of the meat samples affects *Salmonella* heat resistance, possibly due to internalization or attachment (higher surface area) (Orta‐Ramirez et al., 2005). Additionally, chicken skin has a greater fat percentage than chicken thigh meat (47.4% and 10.3%, respectively) and the presence of fat can be protective for *Salmonella* (Juneja, 2001; Murphy et al., 2004). Juneja (2001) found that increasing the fat levels (1 to 12%) in ground chicken samples increased the D‐values and the lag phase times in the 58 to 65°C range. Huang et al. (2019) found that including the fat content as a factor greatly increased the accuracy of regression models for the thermal inactivation of *Salmonella* in meat. This is also reflected in the USDA‐FSIS Time‐Temperature Tables for ready‐to‐eat poultry products (commonly known as Appendix A), where increasing fat content results in increasing cooking time (U.S. Department of Agriculture, Food Safety & Inspection Service, 2017). Whole chicken wings (107 g) have an estimated 13.7 g of fat or about 12% (U.S. Department of Agriculture, Food Safety & Inspection Service, 2018). The fat content is not distributed homogeneously throughout the product, and the areas with higher fat content could increase the heat resistance of *Salmonella* (Rao et al., 2020).

At the treatment level, convection and air frying ovens use hot air and high‐speed air circulation to heat foods from all sides (Zaghi et al., 2019). This leads to superficial dehydration and the gradual formation of a crust on the surface of the food (Zaghi et al., 2019). Moisture loss has been reported for air‐fried protein foods such as chicken nuggets and sardines (Cao et al., 2020; Ferreira et al., 2017). Cao et al. (2020) found that the moisture content of chicken nuggets decreased from 140 g/100 g dry basis to 60 g/100 g dry basis after air frying for 18 min at 180°C (356°F). Air‐fried sardines (10 min at 180°C/356°F) had 55.2 g/100 g of moisture compared with 75.2 g/100 g in the raw product (Ferreira et al., 2017). During treatment of chicken wings, steam escape was observed on both appliances. This loss of moisture from the product and into the environment is important, as the decrease in relative humidity inside the appliance can impact the effectiveness of the thermal treatment. High relative humidity around products reduces undesirable evaporative cooling at the product surface and prevents unwanted concentration of solutes that might lead to the increased thermal resistance of microorganisms (U.S. Department of Agriculture, Food Safety & Inspection Service, 2017). For example, Murphy, Johnson, Marcy, et al. (2001) found that *Salmonella* counts were higher on chicken breast patties processed (177°C/350.6°F) at low humidity (wet bulb temperature of 48°C/118.4°F) than at high humidity (wet bulb temperature of 93°C/199.4°F). However, high humidity would be undesirable in air frying, as it would interfere with the formation of the crust and the “fried” characteristics of the product.

Both the convection and air frying ovens were capable of inactivating high levels of *Salmonella* on chicken wings at the cooking times evaluated. Enumeration studies of *Salmonella* in retail raw chicken parts have found very low numbers of cells (1 to 4 CFU per chicken part), and wings were among the parts more likely to be contaminated (Oscar, 2014; Oscar et al., 2010). However, even low numbers of *Salmonella* can pose significant public health risks if the product is temperature abused, undercooked, or consumed by individuals from a high‐risk population (Oscar, 2014).

## 4 CONCLUSION

There was no difference in *Salmonella* inactivation between convection oven and the air fryer treatments. Similarly, there was no difference between the three different temperatures tested in the air fryer. Therefore, of the three factors considered (appliance, cooking temperature, and cooking time), the most important factor for microbial inactivation was cooking time. The cooking time required for a 7‐log reduction was higher than in the predictions from calculated lethality values, possibly due to the higher complexity of the chicken wings matrix. This study highlights the importance of validating thermal treatments in novel appliances under the conditions of domestic and foodservice use to ensure consumer food safety.

## AUTHOR CONTRIBUTIONS

Carmen Cano: Conceptualization‐Equal; Data curation‐Lead; Formal analysis‐Lead; Methodology‐Equal; Project administration‐Equal; Writing original draft‐Lead; Writing review & editing‐Supporting. Xinyao Wei: Formal analysis‐Equal; Methodology‐Supporting; Visualization‐Lead. Cyril Etaka: Investigation‐Supporting; Methodology‐Supporting. Byron Chaves: Conceptualization‐Equal; Funding acquisition‐Lead; Methodology‐Equal; Project administration‐Equal; Resources‐Lead; Supervision‐Lead; Writing review & editing‐Equal.

## CONFLICT OF INTEREST

None to declare.
